# The Surreptitious Abolition of Compulsory Vaccination

**Published:** 1907-06-22

**Authors:** 


### Public Health and Hygiene.

### THE SURREPTITIOUS ABOLITION OF COMPULSORY VACCINATION.

A Bill to substitute a statutory declaration of conscientious objection for the certificate required under Section 2 of the Vaccination Act of 1898 seems on the face of it a very innocent affair. In essence all that it proposes is to enable the parent to make a statutory declaration that he conscientiously believes that vaccination would be prejudicial to the health of his child. This declaration may be made before a commissioner for oaths at a cost of from 1s. to 1s. 6d., and is to replace the public procedure of satisfying a magistrate that the applicant has a conscientious objection to vaccination, prior to the granting of an exemption certificate. The proposal raises three questions, which at the present juncture we consider of considerable importance.

1. Is it necessary that vaccination be compulsory in order that the nation may be safeguarded from the ravages of small-pox?

2. If it be, should private judgment be so far respected as to exempt objectors under any circumstances from the operation of the law?

3. If it be granted that such exemption may for practical convenience be allowed, may it be made easy of attainment, or be obtainable in private by aid of scheduled forms of declaration and at a lesser degree of trouble and cost than is incidental to vaccination itself?

If the answer to the first question be in the affirmative, the answer to the third one would, we think, most certainly be in the negative. Yet, incredible though it appear, Parliament is asked to answer affirmatively the whole of these questions, and that at the instigation of the President of the Local Government Board, the Minister who is the official representative in the Government of the great interest of the national health. With regard to the first of these questions we have little to say. Authoritative opinion is so overwhelming in the decisiveness of its answer that not even the personal predilections of Mr. John Burns will induce him to ask the House of Commons to declare clearly and definitely that compulsory vaccination is unnecessary. To do so would be too flagrant a defiance of expert opinion. Vaccination must, at least in name, remain compulsory, if even the appearances of decency are to be preserved in Parliamentary government. And since the answer to this question is not in dispute, it were supererogation to discuss it.

When we address ourselves to the second question we find it has the sanctity of recommendation by a Royal Commission. It has the further recommendation of having been found to work on the whole rather better than the method of penalising the conscientious objector, which was the alternative. We confess that our objection to the Act of 1898 is academic, even doctrinaire, rather than practical. Once habilitate conscience as valid ground for exemption from the operation of the law and there is no longer law, but only personal choice. To do so is to set up private judgment as the arbiter of acts which by common decree are forbidden, and to establish a moral sanction for what the law condemns. But indefensible as in principle it is, it may justly be claimed that it has not militated against the practice of vaccination, and we think, therefore, that private judgment may be respected to the extent of exempting objectors from the operation of the law, provided that in effect it is found that such objectors are fewer, or at any rate not more numerous, than the penalisable defaulters whom they replace.

But when we come to consider the third question to which we have addressed ourselves, it seems to be only a roundabout way of repeating the first. That is not compulsory with which it is more difficult to comply than by due process of law to avoid. Yet this is what the Bill which has passed its second reading proposes. Vaccination does mean trouble to the parents: there are the visits to or from the doctor; there is the pain to the mother, through her enduring superstition that vaccination is "cruel" to the child; there is a brief though definite illness as a direct result of the vaccination; and to the large number of people who do not avail themselves of the services of the public vaccinator there is the cost of the vaccination and of its subsequent treatment. To those whose prejudices against vaccination do not amount to "conscientious objection" within the meaning of the Act, to those even who are indifferent to the protection which it affords against what to the parents appears the remote contingency of small-pox, the Bill proposes a simple inexpensive untroublesome escape from the "bother" which vaccination entails. One would have thought that even on grounds of jurisprudence, quite irrespective of public health considerations, it would have been unwise to have weakened respect for the law by providing an escape by a channel easier of following than that which constitutes compliance. The fact is that we have ceased to look upon vaccination as compulsory, and the right to exemption having been recognised, Parliament is intent only on reducing to a simple and uniform procedure the steps by which exemption is to be obtained. The new Vaccination Order just issued by the President of the Local Government Board lays another emphasis on the non-compulsory character of vaccination, such as was never contemplated by the promoters of the Act of 1898. The new form Q may be described as an official advertisement that a "legal excuse" for non-vaccination may and must be obtained within a month of the issue of the notice, or an offer to vaccinate the child will be made by the public vaccinator.

The period during which a "legal excuse for the non-vaccination of the child" may be obtained expires a month after the time at which Form Q must be sent to the parent. Read in conjunction with the proposals of the new Bill, it is impossible to escape the conclusion that the effect will be to make vaccination in the future more honoured in the breach than the observance. The proverb has it that there are more ways of killing a dog than hanging him, and it is a tribute to the mastery of finesse of the astute President of the Local Government Board that with the passage of his Bill he will have effectually abolished, almost without opposition, the compulsory character of vaccination in England and Wales.

To medical men, who best can judge what the effect of this will be, the matter will appear in a wholly different light. They know, as Sir John Batty Tuke said on the motion for the second reading of the Bill, that "as surely as the sun will rise to-morrow, small-pox will reappear in our midst, and work its deadly power among what will have become an unprotected community." This is the persistent, emphatic, and uniform pronouncement of expert and authoritative opinion. It has not been overridden, but those who are prepared to give it legislative effect bid fair to be outflanked in these latest tactics of the antivaccinists.

